# Common hierarchies, varied rules - the problem of governing community first responders in prehospital care for quality standards: documentary discourse analysis

**DOI:** 10.1186/s12913-022-08960-w

**Published:** 2023-01-16

**Authors:** Gupteswar Patel, Viet-Hai Phung, Ian Trueman, Roderick Orner, Aloysius Niroshan Siriwardena

**Affiliations:** 1 Community and Health Research Unit, School of Health and Social Care, Lincoln Medical School, University of Lincoln, Lincoln, LN6 7TS, UK; 2 LMS 3006, Lincoln Medical School, University of Lincoln, 3006, LN6 7TS Lincoln, UK

**Keywords:** Community first responders, Docile bodies, Volunteers, Cardiac arrest, Prehospital emergency care, Policy, Power dynamics

## Abstract

A key focus is placed on engaging communities to become involved in making decisions to support health and care services in healthcare policies in England, UK. An example is the deployment of volunteers such as community first responders (CFRs), who are members of the public with basic life support skills, trained to intervene in emergency situations prior to the arrival of ambulance services. CFR policies have been devised by National Health Service (NHS) Trusts as a way of governing these and related activities. This paper critically examines the discourse around CFR policies to understand how CFR roles are organised and monitoring governance mechanisms are delineated in ensuring quality care delivery. We collected ten CFR policies from six ambulance services. Inductive analysis, guided by Foucault’s theory, enabled the identification of themes and subthemes. We found that Trusts have a common goal to make care quality assurances to regulatory bodies on CFR roles, and this is depicted in common hierarchies of individual responsibilities across Trusts. However, policies that govern approaches to CFRs activity vary. Firstly, the paper highlights institutional approaches to ensuring public safety through the application of organised surveillance systems to monitor CFR activities, and draws parallels between such surveillance and Foucault’s docile bodies. Secondly, the paper discusses how varying rules in the surveillance system compromises safety by decentralising knowledge to regulatory bodies to whom NHS Trusts must make safety assurances. We suggest that stronger interrelationships between Trusts in considering the CFR role has potential to increase public safety and outline a clearer direction for CFRs.

## Introduction

A key focus of the England’s National Health Service (NHS) Five Year Forward Review (2014) [1] was on engaging communities to become involved in making decisions to support future health and care services. The review emphasised the importance of volunteers in rural healthcare settings and devising ways to help volunteers become part of the NHS family, “not as substitutes for but as partners with our skilled employed staff (p 13)”.

Community First Responders (CFRs) are volunteer members of the public who have received basic life support training and, in some cases, additional skills to help ambulance services provide care for people with emergencies. CFRs are often required to respond to immediate life-threatening situations involving heart attack or cardiac arrest [2]. Cardiac arrest is one of the most serious out-of-hospital medical emergencies and usually occurs at the patient’s home or in the community [3]. Rapid resuscitation or defibrillation (administering electric shock) increases the chances of the victim’s survival [3, 4].

Working under designated NHS ambulance organisations or Trusts, CFRs are expected to give basic life support care within their local communities prior to the arrival of ambulance staff [5]. Allowing people with basic training to attend emergency care patients carries with it associated risks [5, 6], and NHS Trusts’ response to such risks is to devise policies and frameworks to govern and guide CFR activities [7]. Examples of such policies are North-West Ambulance Service NHS Trust (2019) and South-Central Ambulance Service NHS Foundation Trust (2020). Existing research has demonstrated that the structure of organisations’ work often reinforces power relations, in which communities and community-level organisations frequently undertake service roles while the governance power is retained and centralised at the mainstream organisations [8]. The study of power relations using Foucault’s work has been extensively researched in healthcare literature furthering our understanding of the role of health professions [9–11], although, the power relations between institutions and volunteer workers, in this case, the ambulance services and the CFRs, remains under-explored. The investigation of these relationships is important in order to understand how CFRs are defined in the health service, how their roles are designed, the systems governing their activities, and the types of development and support services for CFRs. Therefore, we aimed to examine policies, protocols, and guidelines referring to or related to CFRs to comprehend how disciplinary power is entrenched in the structure of the CFR schemes and how embodied hierarchy is reproduced in policy discourse. Disciplinary power is fundamentally ingrained in all relations, which confers the power to transform into disciplined subjects [12]. With this notion, power is crucial for maintaining social order and discipline. Foucault redirected the focus of power away from the governing state towards disciplinary power observed in the administrative arrangements and the system of surveillance and monitoring, which ensured that individuals acquired self-discipline and anticipated behaviours [13].

The investigation of power undertaken in this study builds particularly on the theory of Foucauldian discourse analysis [14]. Foucault used the prison system to demonstrate how institutions use disciplinary power to enforce rules created to resolve ‘problems’ in society, by effectively creating obedient people (or docile bodies) [15, 16]. The concept of a docile body is linked to the notion of discipline and punishment, and was coined by Foucault to illustrates a type of power that instructs people to behave in a specific way, transforming them into docile bodies. These docile bodies are created in a system that is performance-efficient and ‘obedient to authority’ [13]. Docility is also required to control activity of individuals where ‘safety’ of wider society is concerned [15]. The CFR policies included in this discourse analysis were developed with the institutional goals of ensuring patient safety and minimising risk. In this paper, we explore the concept that CFRs are constructed as docile bodies and Ambulance Trusts as disciplining institutions with a group of workforce or docile bodies, through which the Trusts construct normative discipline subjects. Foucault’s concept guides us, highlighting the operation of power and knowledge prevails in CFR policies and emphasises disciplinary actions attributing to infringement of rules in practice.

The object of our analysis focuses on discovering what is represented as the problem in the policies and the types of strategies set out to combat or resolve the problem. We explore both the problem and solution in the context of the institutional approach to discipline and punishment, where rules are set out in strategies to combat the problem, and penalties are applied for non-compliance of the rules. We conceptualise the use of hierarchies to create power dynamics that is able to trace and hold to account those who fail to comply with the policies.

This study aimed to critically examine the CFR related policies and delineate how CFR roles are structured and how policy governance mechanism are formed. Theoretically, this study aimed to establish the relationship between volunteer CFR and ambulance services as regulating agencies by employing Foucault’s concept of docile bodies.

## Methods

### The policy documents and settings

We obtained ten CFR policies, protocols and guidelines from CFR leads and managers in seven Ambulance Trusts across England. These documents were seven CFR policies, one Covid-19 guideline for CFRs, and two protocols developed for specialist CFR roles, namely CFRs attending patients who had fallen, and CFRs who had received additional drug administration training. These seven ambulance services are East Midland Ambulance Service, North West Ambulance Service, South Coast Ambulance Service, South East Coast Ambulance Service, South Western Ambulance Service Foundation, West Midland Ambulance Service, and Yorkshire Ambulance Service, NHS Trusts. These ambulance services are part of the United Kingdom NHS and cover rural areas of England, providing emergency care to people with acute illness and injuries.

### Analysis of the policies

We employed Foucauldian discourse analysis [14, 17] to examine the policy documents. Foucauldian discourse analysis is central to the study of power and focuses on the construction of the meaning of social actions, practices and texts using a lens of power relations by indicating the unwritten rules and structures that produce a set of institutionally and culturally prescribed rules [18]. In general, discourse analysis entails the examination of texts [19], and in this study the texts were policy documents related to CFRs. The analysis focused on how meanings were connected to show a discourse through the keywords and sentences, the corelation between texts, their positioning and functions. For example, what do the keywords say about CFRs’ role in rural healthcare and what else do they show? What are the contradictions, diversities, dispersions, and how do they come together to define purpose of the documents and CFRs’ roles?

The data were manually coded with highlights and research memos. An inductive approach [20] was applied to each document to identify the discourse of power in the structure of the CFR schemes or operational guidelines. We constructed our coding framework based on the texts and keywords in order to understand how the CFR roles were articulated in relation to the ambulance services. We developed codes such as training, equipment, procedure, responsibility, governance, rewards or benefits and safety, and organised the CFR roles and responsibilities relevant to these codes. The above enabled us to categorise emerging discourses pertinent to the research goals, which we subsequently synthesised and classified into two overarching themes: The Problem, and The Solution, which serve as subheadings in the following sections.

## Findings

### The problem – governing CFR roles

The discourse analysis shows NHS Trusts exert much effort to maintain high quality CFR programmes and appreciate the value CFRs bring to prehospital care. However, institutional efforts often encompass policies or rules to control CFR activities or practices through various monitoring strategies.

*The Trust Board acknowledges the positive effect on patient outcomes achievable by a well-managed and effectively governed Community First Responder network. (Source: SECAMB Community First Responder Policy V0.2 2018)*

Only by constant monitoring would Trusts be able to guarantee quality to those who regulate them and provide assurance on care quality. Thus, the problem presented in the policies align to risk or patient safety.

Although many types of CFRs exist in various tiers, discourse or the purpose of policy text pays particular attention to those in the bottom tiers, who have minimal training and who must continually justify their knowledge and competencies. Using nurses as an example, Mackintosh and Sandall (2010) [21] argued that the capabilities of nurses were subordinated and undermined based on their clinical knowledge against other clinical professionals. Hence, the solution to this problem was strategised using protocols and structured communication tools to address the power balance in such power dynamic contexts. To achieve safety as a ‘solution’ to risk, there is justification to set out rules which are presented as protocols, procedures, guidelines and frameworks within which parameters (or scope of practice) can be set for compliance. Rules enable CFRs to practice safely within defined parameters to protect Trusts, the public, and the CFR themselves.

Compliance to rules is paramount where safety is concerned or disciplinary action would usually be taken [22, 23]. The language used to encourage CFRs compliance is sometimes threatening:

*On signing the volunteer agreement CFRs agree to return any equipment issued by YAS should they leave the scheme. Failure to return any equipment, including ID cards is a security risk and will be treated as theft. (YAS Volunteer procedure CFR policy v7.0 2020*

In the following, we draw parallels between the solution and Foucault’s docile bodies in the context of coercive discipline and punishment [15, 16, 22]. We highlight inconsistencies in the rules among the various Trusts to suggest that limited inter-relations between Trusts forces decentralised knowledge and creates vulnerabilities that may itself compromise safety, especially across borders.

### The solution – a system of surveillance that disciplines and punishes

Docile bodies are targets of power that are subjected, used, transformed and improved [15, 24]. Policies for CFRs discuss them as subjects that can be used (for responding to emergencies, or fundraising), transformed (into clinically knowledgeable) and improved (through continuing training) to achieve safe care. Discipline, consisting of techniques of assuring order, is exercised through creation of rules, correct training in acceptable practice, and appointing a hierarchy of monitors - identifiable by rank - that implement, supervise, enforce the rules, and keep a tight control on activities [15, 22, 25, 26]. Discipline creates docile bodies [27]. Safety of CFRs’ work is demonstrated through disciplinary strategies of identifying risks, practising within set parameters, and adhering to an agreed code of behaviour. In documents examined, expectations of CFRs were clearly spelt out with words such as ‘must’, ‘should’, and ‘expected’:

*Responders are expected to provide care up to, but not exceeding, their level of training, contractual scope of practice and competency in order to act in the best interests of the patient. All Responders will receive an initial period of education to ensure they have the knowledge and skills to meet the Trusts quality standard and to provide the best experience to our patients. (SWASFT Responder governance policy v9 2019).*

Self-discipline and individual responsibility are emphasised. In documents examined, CFRs are expected adhere to these responsibilities as individuals (see Table 1).

**Table 1:** Individual responsibilities of community first responders

Identify risks and safety issues associated with CFRs and their work based on quality care standard	• Report criminal charge or conviction immediately. • Report any changes or endorsements to their driving licence • Inform their insurer that their private vehicle is being used for a responding purpose and is liable for any claims when using the vehicle for responding. • Report that they are pregnant at the earliest opportunity.
Set procedures and policies, and framework to address the identified risks and safety issues	• Familiarise themselves with the NHS Constitution and the Trust’s core values. • Ensure that their behaviours reflect Trust’s core values when they are undertaking voluntary activity and representing the Trust. • Ensure equipment is clean and in a serviceable condition when handing over. • Keep equipment and consumables safe and ensuring the equipment is serviceable. • Maintain their vehicle in a safe and roadworthy condition and ensure it is sufficiently covered by suitable motor insurance and MOT. • Be responsible for providing their own defence at their own expense. • Not assess and leave patients on-scene without the clinical responsibility being taken by a responding Clinician or a Clinical Supervisor. • Take sufficient rest and where relevant manage their normal employment commitments and their CFR voluntary time. • Follow the guidelines on attire to respond to minimise the risk of injury to themselves. • Raise with their local manager any perceived deficiencies or lack of contemporary experience in any practice area. • Maintain sufficient fitness for their duties. • Follow guidelines in policies relevant to the CFR.
Setout a chain of command to monitor compliance to the procedure and policies and framework	• Adhere to responsibilities set out in Trust’s code of conduct. • Inform Community and Engagement Training Officer (CETO) through their coordinator when levels of medications are running low. • Use online communication networks sensibly and responsibly and to consider the wider implications of using social networking sites. • Not to breach any confidentiality. • Be aware of their responsibilities toward consumption of alcohol and ensure their alcohol level is zero. • Always adhere to the Trust’s Infection Prevention and Control policy and help ensure continued compliance of the Trust with the Health and Social Care Act (2008) • Comply with Health and Safety standards as set by the Trust. • Ensure that the scope of practice is maintained.
Document the compliance for auditing	• Complete a patient report form • Raise concerns. • Take account of the Working Time Directives. • Report any changes or endorsements to their driving licence. • Ensure that they produce their driving licence, MOT certificate and insurance certificate for ongoing checks. • Report that they are pregnant at the earliest opportunity. • Highlight any items requiring servicing prior to the expiry date. • Report any actual or potential incident using the Trust’s incident reporting procedure.
Use audits to make assurances to governmental bodies that care quality standards are being met	• Ensure that they do not fall outside of requalification period or be asked to stand down. • Report any actual or potential incident using the Trust’s incident reporting procedure.

**Fig. 1:**
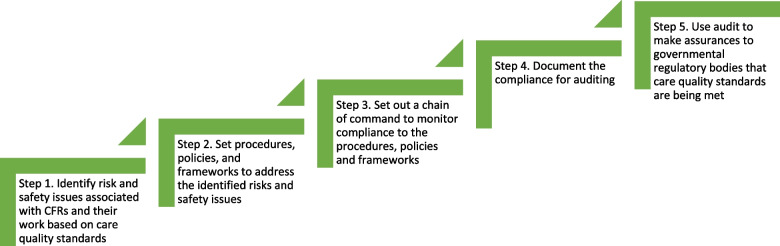
Stairway hierarchy to organised surveillance of CFRs

The discourse analysis reveals these processes in practising safety are a highly organised surveillance system that can expose and track problems, which we compare to steps in a ‘stairway hierarchy’ (Fig. 1). Similar to how compartmentalised spaces are used to hold each individual into account in Foucault’s disciplinary power [15], each step in the surveillance hierarchy is able to separate individual failings and hold to account.

### Identification of Risk

Step 1 in the stairway hierarchy identifies risks and safety associated with enrolling a CFR to administer care. Risk and safety issues identified ranges from the moment a CFR is recruited to after their work ceases. To expose risk associated with the CFR, constant checks are performed. To widen the scope of identifying and mitigating risk, self-discipline is imposed on CFRs themselves who are tasked to be active identifiers of safety issues including in their own behaviours [28, 29]. A CFR must self-report any criminal convictions or driving penalties and act as an advocate for safeguarding patients and their families or carers as well as being given the freedom to speak up about concerns and report incidents. Risk identification is ongoing and dynamic, and can involve acute and chronic issues.

### Implementing rules to address risks

Once risks are identified, step 2 focuses on devising and implementing rules to mitigate or eliminate risk. For every risk or safety issue, there are a number of rules to address the issue. There are rules for attending an emergency call, safeguarding patients, administering care at scene, responding to patients refusing CFR care, handing care over to ambulance service resources, travelling in the CFR’s own car, using a Trust car, and so on. CFRs are trained and expected to follow guidelines and obey rules [22], be competent and practised [30–32]. Rules are however not always consistent, in particular, training programmes, pre-duty routines, investigative procedures, scope of practice, and pathways to development vary between Trusts.

### A chain of command to monitor compliance to procedures

Networks of people who implement and enforce the rules keep CFR activities under tight control. Chiefly consisting of NHS Trusts’ paid workforce, their responsibility is to operationally monitor compliance with the rules. There is a hierarchy within this network which critically appraises problems [25] and channels them to the right ‘Chain of Command’. Each Trust has its own procedural wording, its own list of enforceable actions, and its own hierarchies and job titles for the workforce. Operational responsibilities are shared between the Trust workforce, individual CFRs, and external bodies. However, each Trust’s workforce has overall responsibility in governing compliance with its own procedures in line with regulatory bodies agenda. Government regulatory bodies are at the top of this hierarchy, as responsible persons attached to the various rules cascade down to community-level responsible persons.

### Documenting compliance for auditing

To reveal obstacles and manage waste [25], records of CFR activities are kept for tracking, tracing, and re-tracing of success stories, obstacles, non-compliance to rules, and further identification of risks. Documentation involves recording every aspect of the CFR role from recruitment to termination and all activities in-between. Records are stored on CFRs background and character checks, proficiency assessment results, competency checks, driving records, training records, hours of active duty, patient forms used by the CFR, records of patient refusing a CFRs treatment, behaviour issues, CFR attendance to formal meetings and events, complaints from or against the CFR, and overall code of conduct. At regular intervals, the records are used to appraise each CFR’s docility and utility.

### Making assurances based on audits

Audits provide accountability in surveillance systems [7, 33, 34]. NHS Trusts are able to use records to audit safe measurements and procedures against governmental standards, which enables Trusts to make assurances on care quality and access government resources and maintain insurance and liability cover for CFRs.

## Discussion

The stairway hierarchy shows how surveillance is broken down into steps (or parts) which seek to make monitoring trackable and traceable. Steps in the hierarchy stairway model are strongly interlinked so that they create a trail for every event. Every decision and its responsible personnel can be traced forward and backwards with this approach. The strength of this approach is that goals and expectations are clear and explicit [35–37]. However, the three elements of Foucault’s docile bodies are present in the stairway hierarchy: observation, normalising judgment and examination.

We find that for CFRs to be partners with their skilled colleagues, as depicted in the NHS Five Year Forward Review, they have to undergo a regime of hierarchical observation. CFRs are constantly monitored through various regular checks during their time in the role. Through rules at various levels of the governance hierarchy, CFRs are supervised and controlled, of which parallels can be drawn with Foucault’s ‘classical observatories’ [15, 38].

Foucault argued that the social subjects are created through multiple discursive processes of docile bodies and discipline and punishment [39]. The notion of ‘discipline and punish’ enables researchers to understand the phenomena of organisational structure in constructing human resource policies for ensuring obedience [40]. This paper contributes to demonstrate the extent to which this tendency of docile body and discipline and punishment are involved in the formulation of CFR policies in England. Using penalties as a normalising judgement, control is achieved of individual CFRs and their activities. The policy areas of investigations, suspensions and immediate terminations would be exercised if there are deviations from rules. Finally, examination is used to survey the CFR’s ongoing suitability for the role. CFRs have to undergo constant training and retraining, assessments and re-qualification which are recorded and used to appraise the CFR’s competency for the role.

We found that the variations in rules across NHS Trusts and other organisations decentralises knowledge from the top-national level and suggests little inter-relation between CFR strategies across Trusts. For example ‘Level 1 CFR’ means different things in different Trusts. Trusts have their own training systems, standard rules and penalties. While decentralisation give NHS Trusts power to create their own policies, rules and governance processes, there are potential vulnerabilities in the knowledge it creates. Regulatory bodies are capable of confusing the meaning of a ‘Level 1’ CFR when dealing with different Trusts and CFRs. We suggest this form of decentralisation has a potential to compromise safety, which undermines the highly organised surveillance system, and support the development of national policies, guidance and terminology for the CFR role.

Mackintosh and Sandal suggest that standardising protocols could be an effective means of communication in hierarchical settings, and they utilise a documentation process to illustrate how nurses may still apply their skills to help doctors in hospitals [21]. In our study, the objective of Ambulance Trusts was to provide assurances to regulatory bodies on behalf of CFRs, and managers of CFRs have oversight of implementation of strategies devised to achieve that goal. However, this study highlighted the means of surveillance and compliance in the governance strategies of the institutions.

Previous research has shown that there is no such entity as a “free” body, one that is not subject to surveillance, and that disciplinary power is eternally integrated in the governance system [41]. This study sheds light on the disciplinary power entrenched in CFR policies by examining CFR-related documents. The policies are organised to develop the capacities and duties of CFRs in order for ambulance services to function effectively, with concurrent surveillance to maintain documentation of CFR roles and policy compliance. It can be argued that power relations in the CFR governance were designed to assure compliance with guidelines and that compliance means CFRs become obedient, as highlighted by Gastaldo and Holmes (2002) in their analysis of the nursing profession [11]. Ambulance services operate as the examining mechanism through observations of CFR roles, the manifestation of governance processes, and examination of compliance protocols. The understanding of power in health professions literature has been prominent for decades, however, this study is first of its kind to highlight the power relations between ambulance services as institutions and CFRs as the volunteers.

There is a requirement of a more supportive policy structure for CFR functions when using volunteerism as a strategy of the schemes. Furthermore, we anticipate a supportive framework would strengthen the whole CFR endeavour rather than use of power and surveillance to ensure community engagement in health. A limitation of this study could be attributed to its method of document analysis, which does not provide insight into power dynamics in real-world practices and their implications. In line with this study, future research should explore the power relationships between CFRs, ambulance physicians, and Trusts using primary data.

## Data Availability

The datasets used and/or analysed during the current study are available from the corresponding author on reasonable request.
